# Autophagy-related gene *LRRK2* is likely a susceptibility gene for systemic lupus erythematosus in northern Han Chinese

**DOI:** 10.18632/oncotarget.14631

**Published:** 2017-01-13

**Authors:** Yue-miao Zhang, Xu-jie Zhou, Fa-juan Cheng, Yuan-yuan Qi, Ping Hou, Ming-hui Zhao, Hong Zhang

**Affiliations:** ^1^ Renal Division, Peking University First Hospital, Peking University Institute of Nephrology, Key Laboratory of Renal Disease, Ministry of Health of China, Key Laboratory of Chronic Kidney Disease Prevention and Treatment (Peking University), Ministry of Education, Beijing, People's Republic of China

**Keywords:** autophagy, shared genetics, systemic lupus erythematosus, LRRK2, functional annotation

## Abstract

Autophagy is associated with various immune diseases, including systemic lupus erythematosus (SLE). Seven variants within autophagy-related genes previously reported to show top association signals by genome-wide association studies in immune diseases were selected for analysis. Initially, 510 SLE patients (631 controls) were enrolled in the study. An additional independent cohort of 511 SLE patients (687 controls) was included for replication. Polymorphism rs2638272 in *LRRK2* gene showed significant association with susceptibility to SLE (*P* = 1.14 × 10^−2^) within the initial patient population. This was independently replicated (second patient cohort), and was reinforced with combination (*P* = 2.82 × 10^−3^). By combining multiple layers of regulatory effects, rs1491941 in high linkage disequilibrium with rs2638272 (*r*^2^ = 0.99) was regarded to have the strongest function in *LRRK2*. The rs1491941 protective A-allele exhibited an increase of nuclear protein binding, and an increase in *LRRK2* transcription compared with G-allele. Furthermore, we observed increased transcription levels of *LRRK2* in peripheral blood mononuclear cells from SLE patients compared with controls. In conclusion, we have identified a novel genetic association between the autophagy-related *LRRK2* gene and susceptibility to SLE. By integrating layers of functional data, we derived the beneficial effect of autophagy on the pathogenesis of SLE.

## INTRODUCTION

Systemic lupus erythematosus (SLE) is an autoimmune disease with a strong genetic component [1]. With the development of genome-wide association studies (GWAS), 62 loci have been correlated with susceptibility to SLE [2], which has improved our understanding of the genetic basis of SLE, especially for implicating newer layers of pathways involved in disease pathogenesis [3]. One such pathway is autophagy, which implies a common biological pathway in both autoimmune and autoinflammatory diseases [4, 5].

Autophagy is an endogenous process by which the cell can remove long-lived proteins and damaged organelles through lysosomal degradation. Autophagy has been suggested to be involved in the activation of both innate and adaptive immunity [6]. Previous studies have identified genetic variants within or near *ATG5* (a key autophagy gene required for the formation of autophagosome) by GWAS from SLE patient samples, suggesting that autophagy may be involved in SLE pathogenesis [7, 8]. Furthermore, several other autophagy genes have been identified as susceptibility genes for SLE by recent genetic studies, including *ATG7*, *IRGM*, *DRAM1*, *CDKN1B*, *MTMR3*, and *APOL1* [4, 9–12]. However, our understanding of the link between genotype and autophagy in SLE is still on the horizon. Therefore, the detection of more susceptibility loci will enhance this link, and such endeavors may be centered on novel association signal especially for function signal.

As is the case for many immune diseases, associated autophagy-related genes have been identified by GWAS, such as *NUPR1* [13, 14], *IRGM* [15], *CCL2* [16], *LRRK2* [17], *MTMR3* [18], *IL23R* [19–21], and *NOD2* [22]. Considering the common biological role of autophagy in immune diseases [5, 23, 24], shared genetic studies would be helpful for the validation of associations between these autophagy-related genes and autophagy in SLE. In the present study, we integrated multiple gene annotation datasets, including Haploreg [25], RegulomeDB [26], rSNPBase [27], Blood eQTL [28], and ArrayExpress Archive database [29], to prioritize the plausible functional single nucleotide polymorphisms (SNPs) and genes of the associated SNPs because most of the SLE-associated variants are located in non-coding regions of the genome.

## RESULTS

### Allele association analyses

Seven SNPs within autophagy-related genes previously reported as showing top association signals by GWAS in immune diseases were selected for analysis. A total of 510 patients with SLE (33.7 ± 13.32 years; female/male ratio 6:1) and 631 healthy controls (31.97 ± 8.56 years) were included in analysis. After quality control, one SLE patient and the rs10521209 SNP within *NOD2* were excluded for low genotype rate (< 95%). The remaining 6 SNPs (Table 1) were in Hardy-Weinberg equilibrium in both patients and controls (*P* > 0.05). A second cohort containing 511 SLE patients (32.23 ± 11.85 years; female ratio 7:1) and 687 controls (32.70 ± 8.13 years) were enrolled for validation purposes.

**Table 1:** Association of immune disease susceptibility autophagy-related genes with systemic lupus erythematosus

SNP	Chr.	Position (hg38)	Candidate Gene	Minor Allele	MAF (Case/Control, %)	P	OR (95% CI)	Ref.
rs2201841	1	67228519	*IL23R*	T	29.47/28.37	0.56	1.06	[19–21]
rs11747270	5	150879305	*IRGM*	G	46.56/43.42	0.13	1.14	[15]
**rs2638272**	**12**	**40216081**	***LRRK2***	**G**	**36.74/31.85**	**1.44 × 10^−2^**	**1.24 (1.04–1.48)**	[17]
rs4788084	16	28528527	*NUPR1*	T	26.52/27.10	0.76	0.97	[13–14]
rs991804	17	34260706	*CCL2*	C	41.55/39.54	0.33	1.09	[16]
rs2412973	22	30133642	*MTMR3*	A	36.74/39.38	0.20	0.89	[18]

1. Only associations with statistical significance are presented with 95% CI for ORs.

Abbreviations: 2. Chr: chromosome; MAF: minor allele frequency; OR: odds ratio; Ref: reference; SNP: single nucleotide polymorphism.

Of the 6 SNPs previously reported to be associated with immune diseases, only rs2638272 in the *LRRK2* gene was observed to be associated with SLE in the first patient population (rs2638272G with *P* = 1.14 × 10^−2^; OR 1.24 (95% CI 1.04–1.48) (Table 1). The association between rs2638272 and SLE was marginally replicated (*P* < 0.1) in our second independent population and was reinforced by a meta-analysis (*P* = 2.82 × 10^−3^). Interestingly, the association between rs2638272 and SLE remained significant after multiple corrections (corrected *P* = 1.69 × 10^−2^ using the Bonferroni method on 6 SNPs) (Table 2). To detect a genetic association between rs2638272 and susceptibility to SLE (OR 1.3), a statistical power calculation was utilized and resulted in 85.1% for the combined set of 1020 SLE cases and 1318 control.

**Table 2:** Independent replication and meta-analysis of the associated variant in systemic lupus erythematosus

SNP	Minor Allele	Discovery population (SLE vs. controls, 509/631)			Replication population (SLE vs. controls, 511/687)			Meta-analysis		
		MAF (%)	P	OR (95% CI)	MAF	P	OR (95% CI)	MAF	P	OR (95% CI)
rs2638272	G	1.44 × 10–2	1.24 (1.04–1.48)	35.32/31.88	7.69 × 10–2	1.17 (0.98–1.39)	36.03/31.87	2.82 × 10–3	1.20 (1.07–1.36)

Abbreviations: 1. MAF: minor allele frequency; OR: odds ratio; SNP: single nucleotide polymorphism.

### Functional SNP prediction and validation

In addition to SNP rs2638272, 29 proxy SNPs (*r*^2^ > 0.8) in *LRRK2* gene were extracted, resulting in 30 candidate SNPs for functional annotation. The 30 SNPs showed regulatory effects in the Haploreg v4.1 database: 8 variants were found within regions of promoter histone marks, 18 in regions of enhancer histone marks, 8 in regions of DNase-I hypersensitivity, 2 in regions of protein binding, and 26 in regulatory motif regions in more than one cell type. All proxy SNPs were found in regions of expression quantitative trait loci (eQTL) (Supplementary Table 1). Among the 30 candidate SNPs, rs1491941 showed the most functional hits (44 hits), suggesting it may be the disease-causing variant. In the RegulomeDB database, rs1491941 showed the highest score for regulatory effect (3a, TF binding + any motif + DNase peak) among the candidate SNPs (Table 3). Additionally, rs1491941 was regarded as the regulatory SNP using the rSNPBase database, showing proximal/distal regulation and RNA binding protein mediated regulation effects. The concordance of peaks in rs1491941 suggested that this SNP is likely to influence SLE through mechanisms that regulate *LRRK2* gene expression.

**Table 3:** Detailed annotation information of the SLE-associated single nucleotide polymorphism rs2638272 and its proxies

rs2638272 and proxies (r2 in ASN)	Annotated gene	RegulomeDB score	rSNPBase		cis-eQTL				
			rSNP	Proxy/Distal regulation	GTEx pilot analysis (P value)				Blood eQTL (P value)
					Cells Transformed Fibroblasts	Esophagus Mucosa	Muscle Skeletal	Whole Blood	Whole Blood
rs1491940 (0.82)	LRRK2	3a	no	no		8.40E-06		1.80E-07	5.90E-06
rs2638272 (1)	LRRK2	3a	no	no				5.01E-08	
rs1491941 (0.99)	LRRK2	3a	yes	yes				4.32E-07	5.68E-08
rs2708420 (0.94)	LRRK2	4	no	no				6.26E-08	
rs2638229 (0.84)	LRRK2	4	no	no		8.28E-06		1.93E-07	6.43E-06
rs2404577 (0.80)	LRRK2	5	no	no					
rs2708421 (0.81)	LRRK2	5	no	no	4.80E-06	2.78E-06	1.54E-06	8.04E-09	2.56E-10
rs1491932 (0.97)	LRRK2	5	no	no				6.28E-08	7.37E-06
rs2279535 (0.98)	LRRK2	5	no	no				1.03E-07	7.82E-06
rs2638230 (0.98)	LRRK2	5	no	no				6.82E-08	7.82E-06
rs11612806 (0.80)	LRRK2	6	yes	yes					
rs7969112 (0.80)	LRRK2	6	yes	yes					
rs2708418 (0.93)	LRRK2	6	no	no				1.07E-08	
rs2708423 (0.88)	LRRK2	6	no	no				1.54E-07	
rs2708424 (0.95)	LRRK2	6	no	no				1.04E-07	
rs2638233 (0.95)	LRRK2	6	no	no				6.89E-08	
rs2723259 (0.96)	LRRK2	6	no	no				6.28E-08	7.33E-06
rs2263419 (0.94)	LRRK2	6	no	no				7.29E-08	
rs2263420 (0.98)	LRRK2	6	no	no				8.95E-08	3.64E-07
rs2201141 (0.98)	LRRK2	6	no	no				1.08E-07	
rs7970326 (0.98)	LRRK2	6	no	no				1.27E-06	9.04E-06
rs2708419*	LRRK2	No Data	no	no	-	-	-	-	

Abbreviations: 1. ASN: Asian; eQTL: expression quantitative locus; GTEx: The genotype-tissue expression; rSNP: regulatory single nucleotide polymorphism; SLE: systemic lupus erythematosus; SNP: single nucleotide polymorphism.

2. GTEx pilot analysi (PMID: 25954001), Blood eQTL (PMID: 24013639).

**Figure 1:**
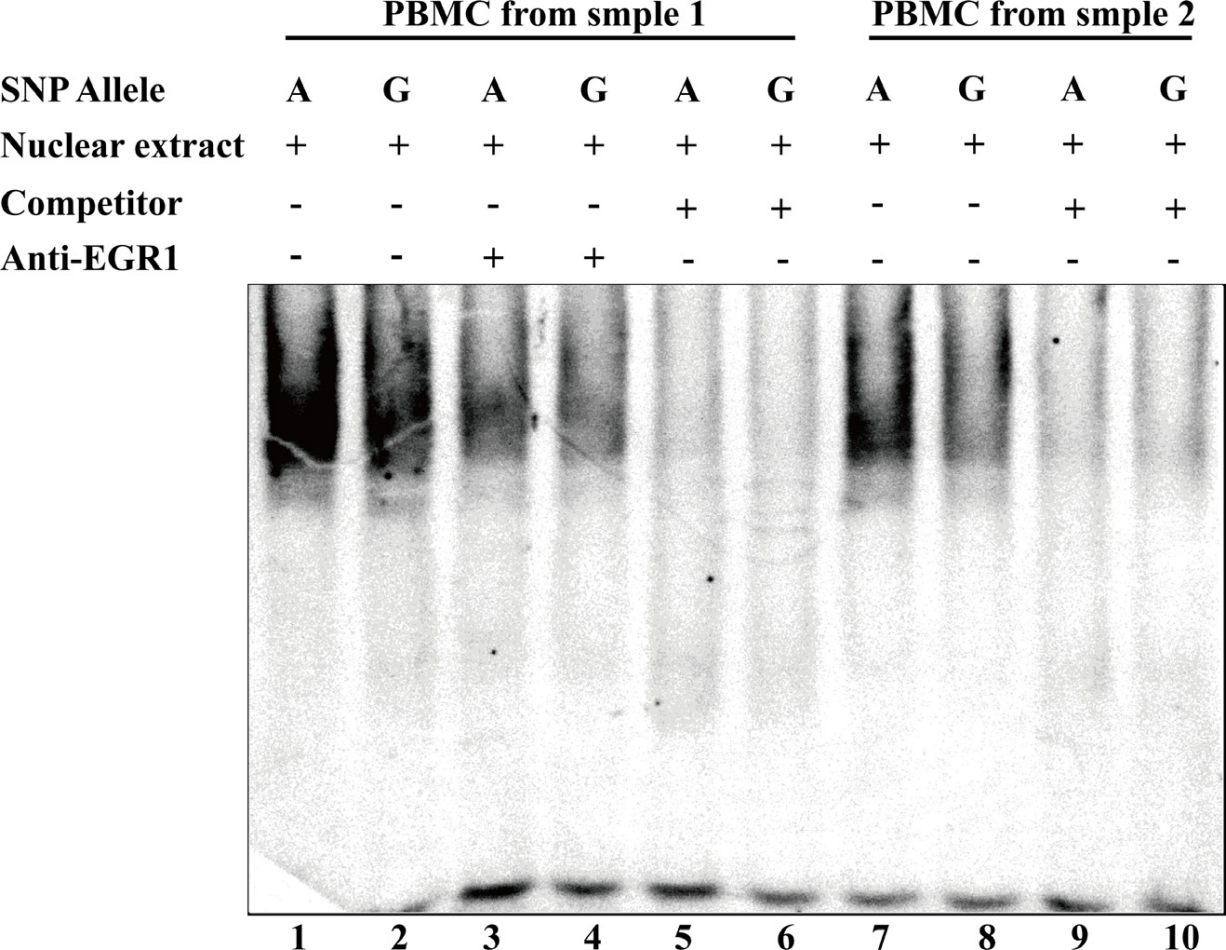
Differential binding affinity of transcriptional factors for the alleles A and G of rs1491941 by electrophoretic mobility shift assay In sample 1, binding affinity of nuclear extract was seen in the order of A > G. Unlabeled probes in 40-fold excess as compared to the labeled probes were used for the competition experiment. Although no significant shift was detected in supershift assays using EGR1 antibody for nuclear extract, the differential binding of unknown nuclear proteins was validated in an independent sample. SNP: single nucleotide polymorphism.

To validate the allele-specific binding affinity of transcription factors to rs1491941, we performed a DNA electrophoretic mobility shift assay (EMSA) using nuclear extracts from peripheral blood mononuclear cells (PBMCs). An increase of transcription factor binding affinity was observed in the rs1491941 A-allele compared with the G-allele (Figure 1); this result was validated independently. In both the Haploreg and RegulomeDB databases, the nuclear protein EGR1 was predicted to bind to rs1491941. However, we performed a supershift assay to examine the binding of EGR1 to rs1491941 but failed to detect a significant shift (Figure 1). Nonetheless, the higher binding affinity for nuclear proteins in the rs1491941 A-allele should increase the transcriptional activity of *LRRK2*.

### Gene expression analyses

We examined lymphoblastoid cell lines from HapMap3 individuals and showed that the A-allele of rs1491941 was associated with an increase of *LRRK2* expression (AA 8.32 + 0.76 among 189 individuals vs. AG 8.24 + 0.79 among 197 individuals vs. GG 8.19 + 0.86 among 48 individuals). However, no significant association between the rs1456896 A-allele and increased *LRRK2* expression was detected (*P* = 0.21), which may due to the small sample size. By searching the Blood eQTL database, which contains data from 5,311 individuals, a significant associated between the rs1491941 A-allele and higher *LRRK2* transcription levels was detected (*P* = 5.68 × 10^−8^, *Z* score = 5.43). Furthermore, the cis-eQTL effect of rs1491941 has been observed in different gene expression profile databases embedded in Haploreg v4.1 (*P* value ranges from 9.04 × 10^−6^ to 2.56 × 10^−10^) (Table 3). Notably, the eQTL effects of rs1491941 were consistently observed in SLE patient blood samples.

**Figure 2:**
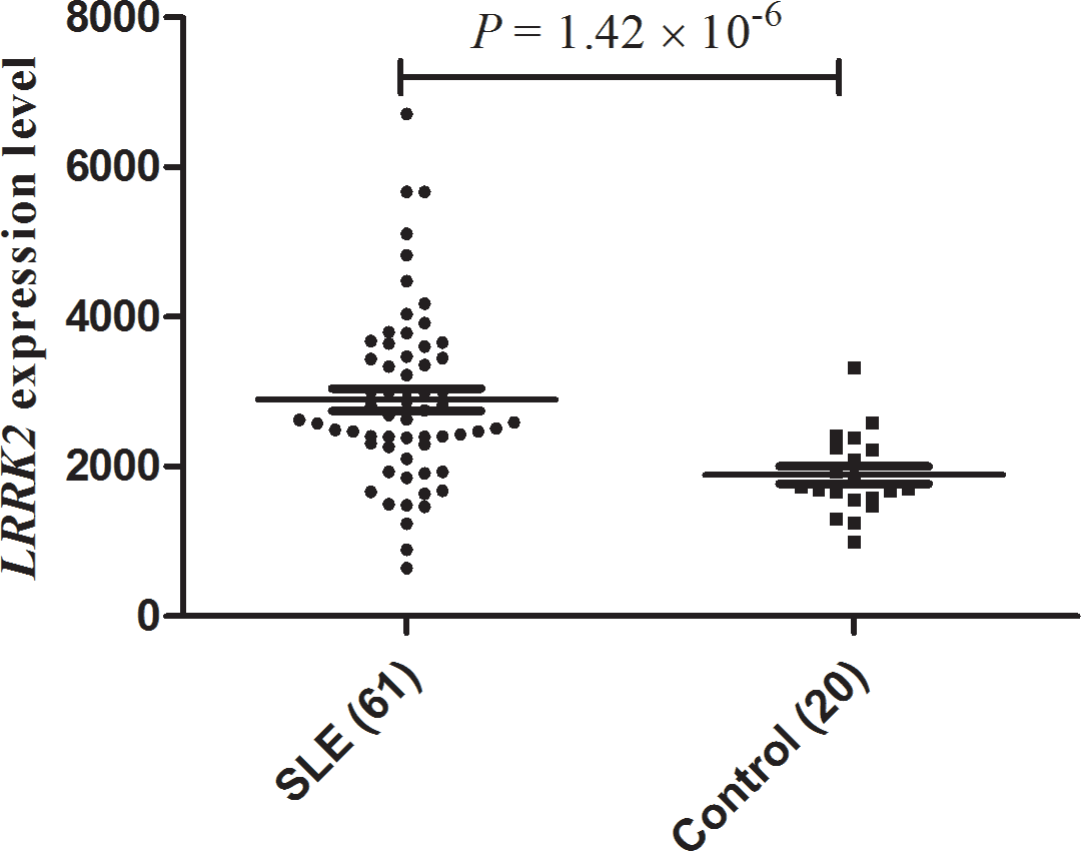
Differential gene expression analysis of LRRK2 in systemic lupus erythematosus patients and controls The figure presents the LRRK2 expression levels in peripheral blood mononuclear cells from 61 SLE patients and 20 normal donor controls (E-GEOD-50772). The expression data of LRRK2 was captured from ArrayExpress Archive database (http://www.ebi.ac.uk/arrayexpress/). SLE: systemic lupus erythematosus.

We observed that *LRRK2* mRNA expression was significantly up-regulated in SLE PBMC compared with normal donor controls (mean + SD normalized fluorescence intensity 2893.56 + 1162.33 among 61 SLE patients versus 1886.76 + 535.16 among 20 normal donor controls; *p* = 1.42 × 10^−6^) (Figure 2).

## DISCUSSION

*LRRK2*, encoding the leucine-rich repeat kinase 2, has been reported to play a role in the autophagy-lysosome pathway [30, 31]. Multiple layers of evidence have suggested that dysfunction of LRRK2 in the immune system may be a central component for the development of immune diseases. For example, increased *LRRK2* expression has been observed in inflamed colonic tissues from patients with Crohn's disease [32]. Furthermore, an analysis of experimental colitis in *LRRK2* KO animals revealed exacerbated disease severity compared with normal animals [33]. Functional studies have suggested that silencing of endogenous *LRRK2* expression results in defects in the induction of autophagy, demonstrating the critical role of endogenous LRRK2 in regulating autophagy [30]. We observed an increase of *LRRK2* mRNA expression in the presence of SNP rs1491941 (A-allele) located in the 3′-UTR of *LRRK2*. In addition, higher *LRRK2* expression levels were observed in SLE patients compared with controls. These findings were consistent with the observation that up-regulated autophagy was observed in both B cells and macrophages in SLE patients [34]. In mice with a lupus-like disease induced by transfer of activated lymphocyte derived DNA, inhibition of macrophageinduced autophagy leads to decreased B-cell maturation and reduced production of dsDNA [35].

However, there are two limitations to the current study. First, we only examined 7 autophagy-related genes that were previously reported to be associated with immune-related diseases by GWAS. Thus, we were not able to systematically assess the genetic contributions of autophagy in SLE. Second, autophagy may exert both beneficial and harmful effects on immunity and inflammation. Although the results from the current study suggest a protective effect of autophagy in the pathogenesis of SLE, additional functional studies are needed to further examine the underlying mechanisms.

In summary, by shared genetic analyses, we have identified a novel genetic association between the variant rs2638272 and susceptibility to SLE. Furthermore, by integrating layers of functional data, we derived the disease-cause variant rs1491941 and revealed a beneficial role of autophagy-related gene *LRRK2* in the pathogenesis of SLE. These results would help broaden our understanding of genetic contributions of autophagy in SLE.

## MATERIALS AND METHODS

### Patients and controls

We recruited 1021 patients with sporadic SLE (diagnosed from 2003 to 2011) and 1318 age-, geographically-matched unrelated controls during the same period from Northern China. All patients fulfilled the criteria of American College of Rheumatology for SLE [36]. All participants provided an informed consent form. The study was approved by the Medical Ethics Committee of Peking University.

### SNP selection and genotyping

In total, 7 SNPs, including rs4788084 in *NUPR1* [13, 14], rs11747270 in *IRGM* [15], rs991804 in *CCL2* [16], rs2638272 in *LRRK2* [17], rs2412973 in *MTMR3* [18], rs2201841 in *IL23R* [19–21], and rs10521209 in *NOD2* [22] were included. Genotyping was conducted using TaqMan allele discrimination assays (Applied Biosystems, Foster City, California, USA).

### Bioinformatic analyses

To prioritize the functional SNPs, we first extracted the proxies (*r*^2^ > 0.8; 1000 Genome project, Asian population was used as reference) for the significantly replicated SNPs using HaploReg v4.1 database (http://www.broadinstitute.org/mammals/haploreg/haploreg.php), which formed the candidate SNPs. Then rSNPBase (http://rsnp.psych.ac.cn/) and RegulomeDB databases (http://www.regulomedb.org/) were searched to examine the regulatory effects of the candidate SNPs. rSNPBase was used to search for regulatory SNPs with experimentally validated regulatory elements controlling transcriptional and post-transcriptional events, whereas, RegulomeDB was used to search for the regulatory scores of SNPs according to their amount of regulatory information (Supplementary Table 2).

The eQTL mapping data in Haploreg v4.1, which includes various eQTL studies, was used for prioritization of the replicated SNPs. Differential gene expression was checked in SLE (E-GEOD-50772) and compared with healthy controls from large-scale genome-wide gene expression analyses (ArrayExpress Archive database; http://www.ebi.ac.uk/arrayexpress/).

### EMSA

For preparation of nuclear extract, the PBMCs from two unrelated healthy blood donors were isolated using lymphocyte separation solution (Ficoll, GE, USA). Then, nuclear extracts were derived from PBMCs using the Nuclear and Cytoplasmic Extraction Reagents Kit (Thermo, USA) according to the manufacturer's instructions. Oligonucleotides (29 base pairs) were designed corresponding to genomic sequences surrounding SNP rs1491941. Single-stranded oligonucleotide probes were labeled using the DIG Gel Shift Kit (Roach, Germany), and sense and antisense oligonucleotides were then annealed. DNA-protein interactions were detected by using a DIG Gel Shift Kit (Roach, Germany) according to the manufacturer's instructions. EGR 1 antibody (ab55160, abcam, USA) was used for supershift analysis. The DNA-protein complexes were separated on a non-denaturing 5% polyacrylamide gel in 0.5 × Tris-borate-EDTA (TBE) running buffer for 120 min at 50 V. The gel was transferred to a nitrocellulose membrane, and signals were detected using a LAS-4000 Image analyzer (GE, USA).

### Statistical analyses

Only SNPs and individuals meeting the quality control criteria of less than 5% overall missing data were included. Testing for deviations from Hardy-Weinberg equilibrium (HWE) was conducted using a χ^2^ goodness-of-fit test separately for cases and controls in different cohorts. Allele frequencies were compared between cases and controls using the χ^2^ test. Statistical analyses were performed with SPSS 12.0 software (SPSS Inc., Chicago, IL). A two-tailed *P* value of less than 0.05 was considered statistically significant. Genetic power was estimated using the Power and Sample Size Calculations Version 3.0 (http://biostat.mc.vanderbilt.edu/PowerSampleSize) with a two-sided type I error rate of 0.05.

## SUPPLEMENTARY MATERIALS FIGURES AND TABLES



## Abbreviations

EMSA: Electrophoretic mobility shift assay
eQTL: expression quantitative trait loci
GWAS: genome-wide association studies
HWE: Hardy-Weinberg equilibrium
PBMCs: peripheral blood mononuclear cells
SNPs: single nucleotide polymorphisms
SLE: systemic lupus erythematosus
