# Supplementary material for: Autophagy-related gene LRRK2 is likely a susceptibility gene for systemic lupus erythematosus in northern Han Chinese
Source: Oncotarget. 2017 Jan 13;8(8):13754–61. doi: 10.18632/oncotarget.14631 (PMC5355135; doi:10.18632/oncotarget.14631)
Supplement: Supplementary file 1 [file oncotarget-08-13754-s001.pdf]

Autophagy-related gene *LRRK2* is likely a susceptibility gene for systemic lupus erythematosus in northern Han Chinese

Supplementary Materials

Supplementary Table 1: HaploReg v4.1 annotations for variants in strong linkage disequilibrium with rs2638272 in Asian population. See Supplementary_Table_1

Supplementary Table 2: The scoring standard of RegulomeDB database

Score	Supporting data
1a	eQTL + TF binding + matched TF motif + matched DNase Footprint + DNase peak
1b	eQTL + TF binding + any motif + DNase Footprint + DNase peak
1c	eQTL + TF binding + matched TF motif + DNase peak
1d	eQTL + TF binding + any motif + DNase peak
1e	eQTL + TF binding + matched TF motif
1f	eQTL + TF binding / DNase peak
2a	TF binding + matched TF motif + matched DNase Footprint + DNase peak
2b	TF binding + any motif + DNase Footprint + DNase peak
2c	TF binding + matched TF motif + DNase peak
3a	TF binding + any motif + DNase peak
3b	TF binding + matched TF motif
4	TF binding + DNase peak
5	TF binding or DNase peak
6	other

Abbreviations: eQTL: expression quantity lucus; TF: transcriptional factor.
